# Prognostic assessment of T‐cells in primary colorectal cancer and paired synchronous or metachronous liver metastasis

**DOI:** 10.1002/ijc.35252

**Published:** 2024-11-07

**Authors:** Andriy Trailin, Esraa Ali, Wenjing Ye, Sergii Pavlov, Lenka Červenková, Ondřej Vyčítal, Filip Ambrozkiewicz, Petr Hošek, Ondřej Daum, Václav Liška, Kari Hemminki

**Affiliations:** ^1^ Laboratory of Translational Cancer Genomics, Biomedical Center, Faculty of Medicine in Pilsen Charles University Pilsen Czech Republic

**Keywords:** colorectal cancer, survival, synchronous and metachronous liver metastases, tumor‐infiltrating lymphocytes

## Abstract

Prognostic value of T‐cells between primary colorectal cancer (pCRC) and its paired synchronous and metachronous liver metastasis (LM) is underinvestigated and is the subject of the present study. We enrolled into this retrospective cohort study patients, who underwent resection of both pCRC and synchronous LM (*N* = 55) or metachronous LM (*N* = 44). After immunohistochemical staining for CD3+, CD8+, and CD45R0+ whole slides were scanned and T‐cell densities were quantified using QuPath software in tumor center (TC), inner margin (IM), outer margin (OM), and peritumor zone (PT) of pCRC and LM. High densities of CD8+ T‐cells in TC, OM and PT of synchronous LM were associated with longer disease‐free survival (DFS). Greater densities of CD3+ T‐cells in IM and PT and CD8+ T‐cells in IM, OM and PT in synchronous LM over pCRC were associated with longer DFS. Greater densities of CD8+ T‐cells in the TC and IM and CD3+ T‐cells in the IM of pCRC were found in the metachronous over synchronous group. The first novel finding demonstrated that high density of CD8+ T cells in synchronous LM were associated with favorable outcome. The second finding of high CD8+ cell density in pCRC in metachronous over synchronous CRC may provide a mechanistic basis for the delay of metastatic spread. Both findings could be applied clinically with own reference values.

## Graphical abstract

What's new?

Whether colorectal cancer with synchronous and metachronous liver metastases represent two distinct phenotypes of the disease remains unclear. This retrospective cohort study demonstrates that tumor‐infiltrating T cells contribute to different clinical courses and prognoses. In patients with synchronous liver metastases, high densities of CD8+ T cells in metastases were associated with favorable outcomes. In patients with metachronous liver metastases, greaterdensities of CD8+ T cells in primary colorectal cancer may be responsible for the delayed metastases. Evaluation of tumor‐infiltrating T‐cells in both primary colorectal cancer and liver metastases could help refine prognosis.

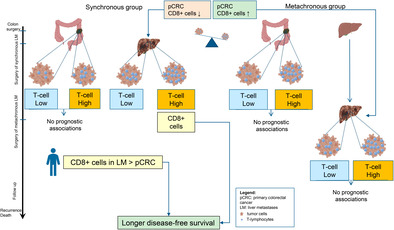

## 1 INTRODUCTION

Colorectal cancer (CRC) is the third most common type of cancer worldwide^1^ with approximately 1.9 million new cases diagnosed annually. CRC also is the second leading cause of cancer‐related mortality with 930,000 CRC‐related deaths annually in the world.^2^ Distant CRC metastases, which is a hallmark of a stage IV of the disease, drastically worsen the survival.^3^, ^4^, ^5^ Up to 25% CRC patients have liver metastases (LM) at the time of diagnosis of primary tumor (synchronous metastases),^6^, ^7^, ^8^ and up to 30% patients at stages I–III develop LM later in course of the disease (metachronous metastases).^7^, ^8^, ^9^ It was suggested that both diseases might represent distinct phenotypes in which synchronous disease shares more aggressive clinical and pathological characteristics,^7^, ^8^, ^10^, ^11^ although not all studies confirmed that.^12^ Surgical resection is the widely accepted first‐line treatment option and the only curative approach for LM.^13^ Nevertheless, the recurrence of the disease occurs in up to 70% of patients^14^ with a 5‐year survival ranging between 20% and 60%.^6^, ^15^, ^16^ Clinical variables have failed to explain survival differences after liver resection.^17^ Clarifying whether a biological difference between the two groups of LM or their primaries exists could have important implications for clinical practice.

In the past two decades, it has become clear that the clinical outcome of CRC does not depend only on the properties of the tumor but also on immune microenvironment, especially on T cells.^15^, ^18^ These include CD3+ pan T cells, effector T cells expressing CD8, and memory T lymphocytes, characterized by expression of CD45RO.^18^ CD3+, CD8+, and CD45RO+ T cells in the tumor center and in the advancing margin of primary CRC stages I–III have overcome other prognostic predictors of survival, including TNM.^18^, ^19^ Prognostic performance of T cells was also demonstrated in LM of stage IV CRC patients,^20^, ^21^, ^22^, ^23^ helping to improve prognostication in this heterogeneous cohort. Only few studies directly compared prognostic abilities of T cells in primary versus metastatic lesions^22^, ^24^, ^25^ with conflicting results. A comparative description of T cells in pCRC and LM in synchronous and metachronous disease is also lacking. Literature is discrepant regarding selection and definition of intratumoral and peritumoral regions of interest (ROI) for the assessment of tumor‐infiltrating lymphocytes.^22^, ^23^, ^26^, ^27^, ^28^

In this paper we outline the abundance and fine regional distribution of T cells from primary CRC to LM in the same patients in order to explore such unresolved questions as: Whether tumor‐infiltrating T cells contribute to different clinical course and prognosis in patients with synchronous and metachronous CRC, and whether prognostic implication of T cells in pCRC is different from T cells in LM? How the immune infiltrates of pCRC and LM are related to each other and is it possible to predict the immunological profile of LM by analyzing pCRC in synchronous and metachronous disease? Does distribution of T cells among different ROIs of pCRC and LM correlate with their prognostic impact?

## 2 MATERIALS AND METHODS

### 2.1 Patients

All consecutive patients undergoing a curative‐intent resection of pCRC followed by liver resection for the first recurrence of CRC in Pilsen University Hospital between 1999 and 2021 were retrospectively identified from hospital archives. Patients with LM detected at the time of diagnosis of primary CRC were qualified as stage IV, synchronous group (*n* = 80). Stage I‐III patients (*n* = 100), in whom LM were detected after resection of the primary tumor, were defined as metachronous group.^29^ Metachronous metastases were detected 1–59 months (median 17 months) following resection of primary tumor.

Patient inclusion criteria in the study were: only first LM, curative intent resection of pCRC and LM, availability of complete clinical and survival information, and availability of good quality formalin fixed‐paraffin embedded tissue (FFPE) of pCRC and LM. Patients with multiple primary neoplasms, with presence of preoperative extrahepatic metastases, history of prior liver resections, and patients who had received neoadjuvant chemoradiotherapy before pCRC surgery, who had undergone emergency surgery, or who had died within 30 days of surgery were excluded from the study. The final cohort consisted of 99 patients (55 stage IV and 44 stages I–III).

Selected information was extracted from the archives and medical files of the patients to record basic demographic, pathological, and clinical data (Table S1).

Pathology reports were reviewed. The clinical stage of the tumor was determined according to the eighth edition of the American Joint Commission on Cancer.^30^ Most patients in both groups had pCRC of histological type NOS and grade 2. Groups of patients with synchronous and metachronous metastases differed only in terms of median size of LM, which was greater in metachronous group and proportion of patients who received FOLFOX chemotherapy, which was lower in metachronous group (Table S1). This retrospective study was conducted in accordance with the ethical standards set out in the Declaration of Helsinki (2013 version); it was approved by the Ethics Committee of the Faculty of Medicine and the University Hospital in Pilsen (300/2020, June 17, 2020).

### 2.2 Pathology and immunohistology

FFPE tissues of pCRC and LM from each patient were identified and cut into 4‐μm sections. In case of multiple LMs, we selected the metastatic tumor with the least regressive changes.^20^ One or two tissue sections were mounted onto BOND Plus Microscope Slides (Cat# 00270, Leica Biosystems Newcastle Ltd., Newcastle, UK). Immunohistochemical detection of CD3+, CD8+, and CD45RO+ T cells was performed using fully automated BOND‐III IHC/ISH stainer. Ready‐to‐use monoclonal primary antibodies for CD3 (clone LN10), CD8 (clone 4B11), and CD45RO (clone UCHL1) all from Leica Biosystems (Newcastle Ltd., United Kingdom) were used. Binding of primary antibodies with their targets was visualized using horseradish peroxidase (HRP)‐linker antibody conjugate system (Bond™ Polymer Refine Detection). Sections were counterstained with Mayer's haematoxylin and embedded into Micromount mounting medium (Leica Biosystems Newcastle Ltd., United Kingdom). Appropriate positive (tonsils) and negative tissue control samples were used throughout.

### 2.3 Image analysis

Whole‐slide scans were obtained using Olympus VS200 scanner (Olympus, Shinjuku, Japan). An open‐source software QuPath v.0.3.2 was used to annotate tumor center (TC), inner margin (IM), outer margin (OM), and peritumor zone (PT) as ROI,^31^ and for image analysis. Before image analysis of pCRC, all lumina, luminal surface of the tumor, large vessels, normal mucosa, dysplastic epithelium, mucosa‐associated lymphoid tissue, muscularis propria, and supportive stroma were excluded.^32^ In LM we excluded all lumina, intervening normal hepatocytes, large fibrous areas or severe regressive hyalinosis without tumor cells, large vessels and bile ducts. Extracellular mucin, fat, necroses, abscesses, hemorrhages, and artifacts were excluded throughout. Density of CD3+, CD8+, and CD45RO+ cells was estimated as the number of immunopositive cell profiles divided by the total area of ROI. To eliminate skewness in the distribution, we converted the raw data into corresponding percentile values and categorized them into low (below 25th percentile) versus high (25th–100th percentile).

### 2.4 Follow‐up

Patients were followed until December 2023, with a median observation time after liver metastasectomy of 84 months (95% CI: 5–163 months) in the synchronous group and 61 months (95% CI: 54–68 months) in metachronous group. Patients were followed up every 3 months for the first 2 years after liver metastasectomy and semiannually thereafter, which included measurement of oncomarkers, chest radiography, abdominal sonogram, and computerized tomography. Positron emission tomography or magnetic resonance imaging were used at the multidisciplinary team's discretion.

### 2.5 Outcomes

We first compared survival since date of colon surgery in synchronous and metachronous groups. The day of liver surgery was chosen then as reference date to compare outcomes between synchronous and metachronous groups. The primary endpoint was disease‐free survival (DFS) that was considered as the time from resection of LM to the date of diagnosis of recurrence or death from any cause. Secondary outcomes were time to recurrence (TTR) and overall survival (OS). TTR was defined as the interval from the date of liver metastasectomy to the date of diagnosis of any site of recurrence. The appropriate proportion of patients without recurrence was denoted as recurrence‐free proportion. OS was defined as the interval from the date of liver metastasectomy to the date of death from any cause. Patients without relapse or death were censored at their last follow‐up.

### 2.6 Statistical methods

We compared cell densities (1) between different ROIs within pCRC or LM, (2) between respective ROIs of pCRC and LM, (3) between respective ROIs of pCRC or LM of synchronous and metachronous groups (Figure S1). We evaluated prognostic impact of factors related to the patient, primary and metastatic tumors for DFS, TTR, and OS. In addition to prognostic impact of individual immune cell types in each ROI of pCRC and LM we tested differences in DFS, TTR, and OS between patients in whom cell densities in LM were greater or smaller compared with pCRC.

Continuous non‐normally distributed data are expressed as median (min–max); their comparison was made either by Mann–Whitney *U*‐test or by Friedman ANOVA, followed by Wilcoxon matched pairs test. Proportions are expressed as raw data (percentages). The associations between pairs of ordinal or quantitative variables were assessed using Spearman correlation due to nonparametric distribution of most of the variables. DFS, TTR, and OS were estimated using the Kaplan–Meier method and compared between groups with the log‐rank test. To determine the prognostic value of individual predictors for TTR, DFS, and OS, Cox regression analysis was performed. GraphPad Prism 9.0 (GraphPad Software LLC) and R software environment were used for the statistical analyses. Survival analysis was performed in the R environment with the Finalfit package.^33^ Kaplan–Meier analysis was performed with the survival package and plots were generated with the survminer package.^34^, ^35^ A two‐sided *p* value <0.05 was considered statistically significant.

## 3 RESULTS

### 3.1 Demographics of CRC patients and outcomes

The demographics, clinical and pathological characteristics of the patients are shown in Table S1.

Patients from the metachronous group showed significantly longer DFS, TTR, and OS after pCRC surgery compared to synchronous group (Figure S2). However after LM surgery, DFS, TTR, and OS were not statistically different between groups (data not shown).

DFS probabilities at 3 years after LM surgery were 11.4% in the synchronous and 17.6% in metachronous groups (Table S2). Median DFS was 10 months and OS 40 months in the synchronous group and 12 and 47 months in metachronous group.

Among 47 and 38 patients, who developed recurrence in the synchronous and metachronous groups the most frequent locations were the liver and lungs (Table S3).

Amongst clinical and pathology variables only older age was associated with longer DFS and TTR and only in metachronous group (Table S4).

### 3.2 Distribution of T cells in pCRC and LM

**FIGURE 1:**
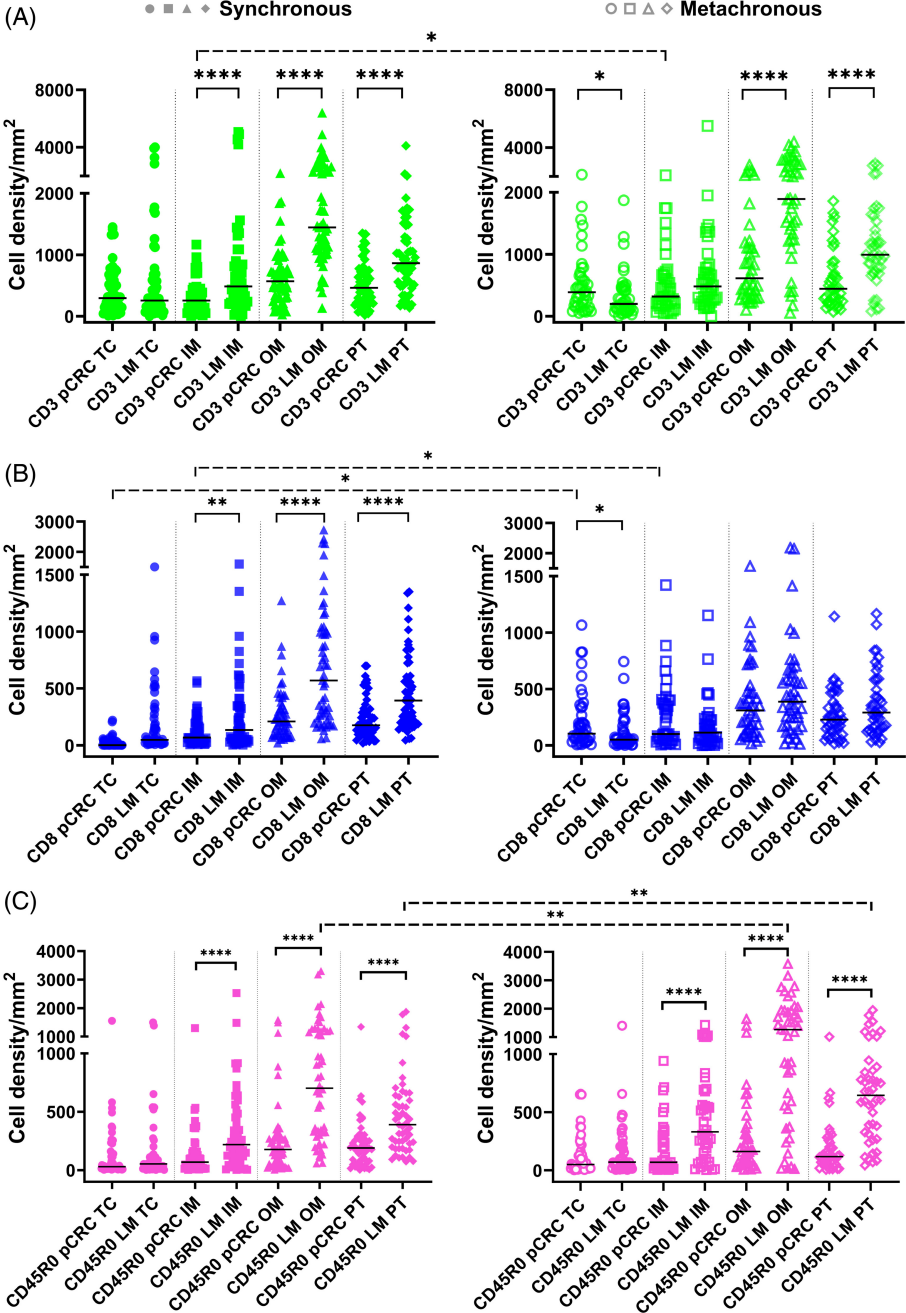
Statistics depicting the spatial distribution of CD3+, CD8+, and CD45RO+ tumor infiltrating lymphocytes per mm^2^ of the section in the TC, IM, OM, and PT of pCRC and LM in patients with synchronous (left panel, filled symbols) and metachronous disease (right panel, empty symbols). Black lines: medians. *p < 0.05, **p < 0.01, ****p < 0.0001. IM, inner invasive margin; LM, liver metastases; OM, outer invasive margin; pCRC, primary tumor; PT, peritumor zone; TC, tumor center.

We compared densities of T cells between pCRC and LM of synchronous and metachronous groups (Figure 1). CD3+ cell densities in both groups were greater in LM compared to pCRC in OM and PT, and additionally in synchronous IM (Figure 1A). For metachronous group, density of CD3+ cells was greater in pCRC compared to LM in TC. For CD8+ cells greater densities in synchronous group were found for LM compared to pCRC in all ROIs (but TC) whereas in metachronous group pCRC had greater densities in TC (Figure 1B). CD45RO+ densities were greater in LM than pCRC in ROIs other than TC (Figure 1C).

We compared then densities of T cells between synchronous and metachronous groups. Although the overall density distributions of T cells were almost similar between synchronous and metachronous groups, greater densities of a few cell types in specific ROIs of metachronous tumors were found as marked with dashed significance bars in Figure 1.

When compared between ROIs, greater densities of all T cells were found in OM and PT compared to the TC and IM in both pCRC and LM as well as in synchronous and metachronous groups (*p* < 0.05 for most comparisons).

Percentage of CD8+ T cells in different ROI of pCRC and LM ranges between 28% and 45% of CD3+ T cells (Table S5).

Correlation analysis of densities of T cells between ROIs of pCRC and LM is shown in Table 1. CD3+ and CD8+ T cell densities correlated only in the metachronous group; for both a concordant correlation was found for TC and some discordant ROIs. Densities of CD45RO+ cells correlated between pCRC and LM in synchronous group only; concordant ROIs were OM and PT.

**TABLE 1:** Spearman correlation between T cells in primary and metastatic sites in CRC patients.

	LM				
	Synchronous				
		TC	IM	OM	PT
pCRC	CD3				
	TC	0.17^a^	0.20	0.13	0.08
	IM	0.14	0.16	0.22	0.13
	OM	0.16	0.23	0.22	0.10
	PT	0.17	0.27	0.14	0.08
	CD8				
	TC	0.06	0.08	0.02	−0.01
	IM	0.11	0.16	0.05	0.04
	OM	0.10	0.21	0.21	0.16
	PT	0.09	0.20	0.11	0.12
	CD45RO				
	TC	0.12	0.02	0.38**	0.31*
	IM	0.1	−0.01	0.41**	0.27
	OM	0.1	0.03	0.40**	0.34*
	PT	−0.09	−0.04	0.38**	0.37**

	Metachronous				
		TC	IM	OM	PT
	CD3				
	TC	0.41**	0.27	0.32*	0.45**
	IM	0.47**	0.33*	0.24	0.38*
	OM	0.30	0.21	0.18	0.40**
	PT	0.20	0.15	0.05	0.24
	CD8				
	TC	0.41**	0.29	0.24	0.32*
	IM	0.42**	0.29	0.15	0.26
	OM	0.34*	0.27	0.26	0.51**
	PT	0.19	0.19	0.01	0.17
	CD45RO				
	TC	0.1	0.18	−0.01	0.09
	IM	0.05	0.16	0.11	0.1
	OM	0.1	0.18	0.19	0.18
	PT	0.18	0.19	0.09	0.14

Abbreviations: CRC, colorectal cancer; IM, inner margin; LM, liver metastases; OM, outer margin; pCRC, primary colorectal cancer; PT, peritumor zone; TC, tumor center.

^a^ Spearman's *ρ*.

* Spearman's *ρ* <0.05;

** Spearman's *ρ* <0.01.

The proportion of patients with greater cell densities in LM versus pCRC (LM > pCRC) versus (LM < pCRC) was compared between synchronous and metachronous groups (Table S6). Greater densities in LM of the synchronous group were found more frequently for CD3+ T cells in OM (*p* = 0.013), CD8+ T cells in IM (*p* = 0.046), and OM (*p* = 0.006).

### 3.3 Association between cell densities and survival

**FIGURE 2:**
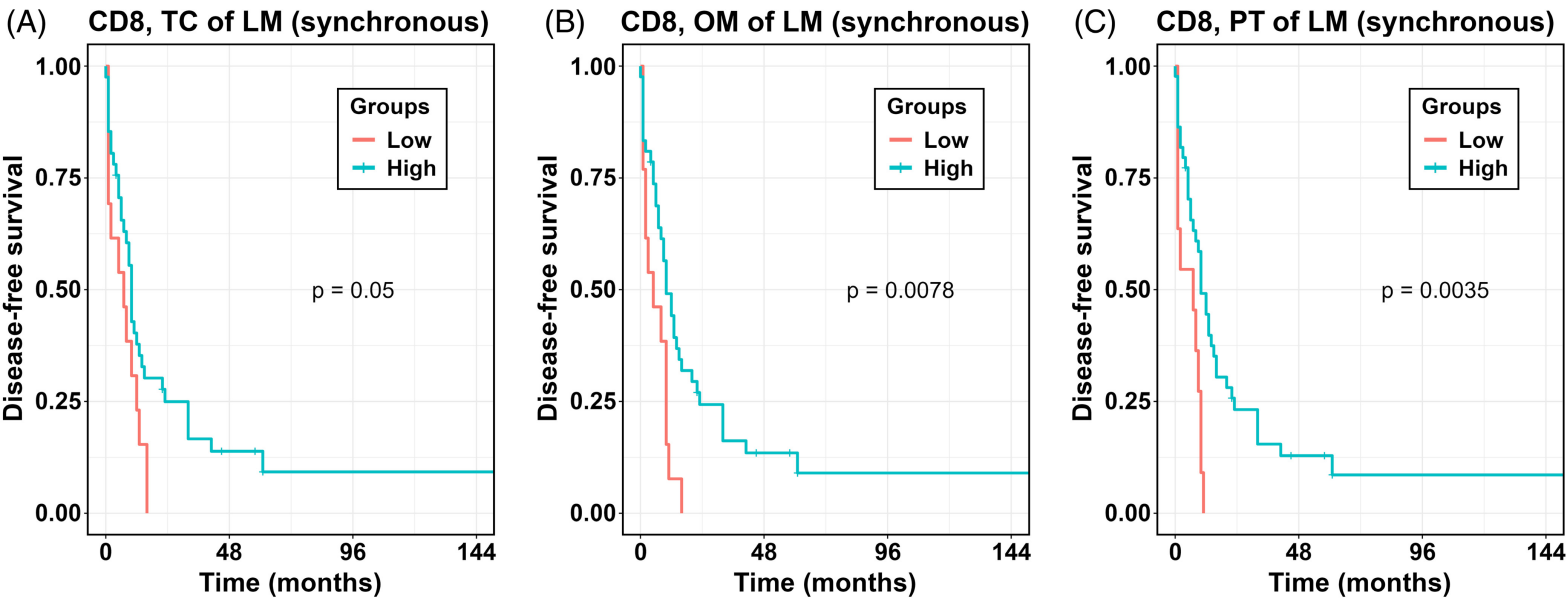
Kaplan–Meier analysis for DFS according to high versus low densities of T cells per ROI. *p* values according to log‐rank test. LM, liver metastases; OM, outer invasive margin; PT, peritumor zone; TC, tumor center.

We first analysed the associations between T cell densities in individual ROI with outcomes and found that high densities of CD8+ T cells in LM (TC, OM, and PT) of synchronous group were associated with a longer DFS; no associations were found for metachronous group (Figure 2, Table 2). Similar associations were seen for TTR (Table S7) and OS (Table S8). Importantly, densities of no cell type in any ROI of pCRC were associated with outcomes in synchronous or metachronous groups (data not shown).

**TABLE 2:** Hazard ratios for DFS between high versus low T cell density per individual ROI of LM in CRC patients with synchronous and metachronous metastases.

Cell type and location	Synchronous, *N* = 55		Metachronous, *N* = 44	
	*N* (%)	HR (95% CI), *p*‐value	*N* (%)	HR (95% CI), *p*‐value
CD3 TC	41 (75.9)	0.68 (0.35–1.31), *p* = 0.244	32 (72.7)	1.13 (0.56–2.28), *p* = 0.740
CD3 IM	40 (74.1)	1.09 (0.55–2.16), *p* = 0.801	33 (75.0)	1.13 (0.54–2.33), *p* = 0.749
CD3 OM	38 (69.1)	0.76 (0.42–1.39), *p* = 0.377	36 (81.8)	0.66 (0.30–1.49), *p* = 0.320
CD3 PT	37 (67.3)	0.76 (0.41–1.39), *p* = 0.369	36 (83.7)	0.51 (0.22–1.21), *p* = 0.128
CD8 TC	**41 (75.9)**	**0.52 (0.27–1.00), *p* = 0.050**	32 (72.7)	0.81 (0.40–1.63), *p* = 0.549
CD8 IM	43 (79.6)	0.70 (0.35–1.41), *p* = 0.318	30 (68.2)	0.94 (0.48–1.85), *p* = 0.865
CD8 OM	**42 (76.4)**	**0.40 (0.20–0.79), *p* = 0.008**	32 (72.7)	0.81 (0.40–1.64), *p* = 0.564
CD8 PT	**44 (80.0)**	**0.34 (0.16–0.70), *p* = 0.004**	30 (68.2)	0.78 (0.40–1.53), *p* = 0.470
CD45RO TC	39 (75.0)	0.96 (0.49–1.87), *p* = 0.901	33 (75.0)	0.82 (0.39–1.71), *p* = 0.598
CD45RO IM	39 (75.0)	1.00 (0.52–1.95), *p* = 0.992	33 (75.0)	0.91 (0.44–1.88), *p* = 0.796
CD45RO OM	37 (69.8)	1.36 (0.71–2.59), *p* = 0.352	36 (81.8)	0.66 (0.30–1.47), *p* = 0.312
CD45RO PT	37 (69.8)	1.29 (0.68–2.45), *p* = 0.444	36 (81.8)	0.94 (0.41–2.14), *p* = 0.874

*Note*: Densities of CD3+, CD8+, and CD45RO+ T cells per area ROI (mm^2^) were converted into percentiles and then categorized into low (0–24 percentile) and high (25–100 percentile). Hazard ratios shows the relative risk compared with 1.00 for the low density. Bold values indicate statistical significance at the *p* < 0.05 level.

Abbreviations: CI, confidence interval; CRC, colorectal cancer; DFS, disease‐free survival; HR, hazard ratio; IM, inner margin; LM, liver metastases; OM, outer margin; PT, peritumor zone; ROI, region of interest; TC, tumor center.

**FIGURE 3:**
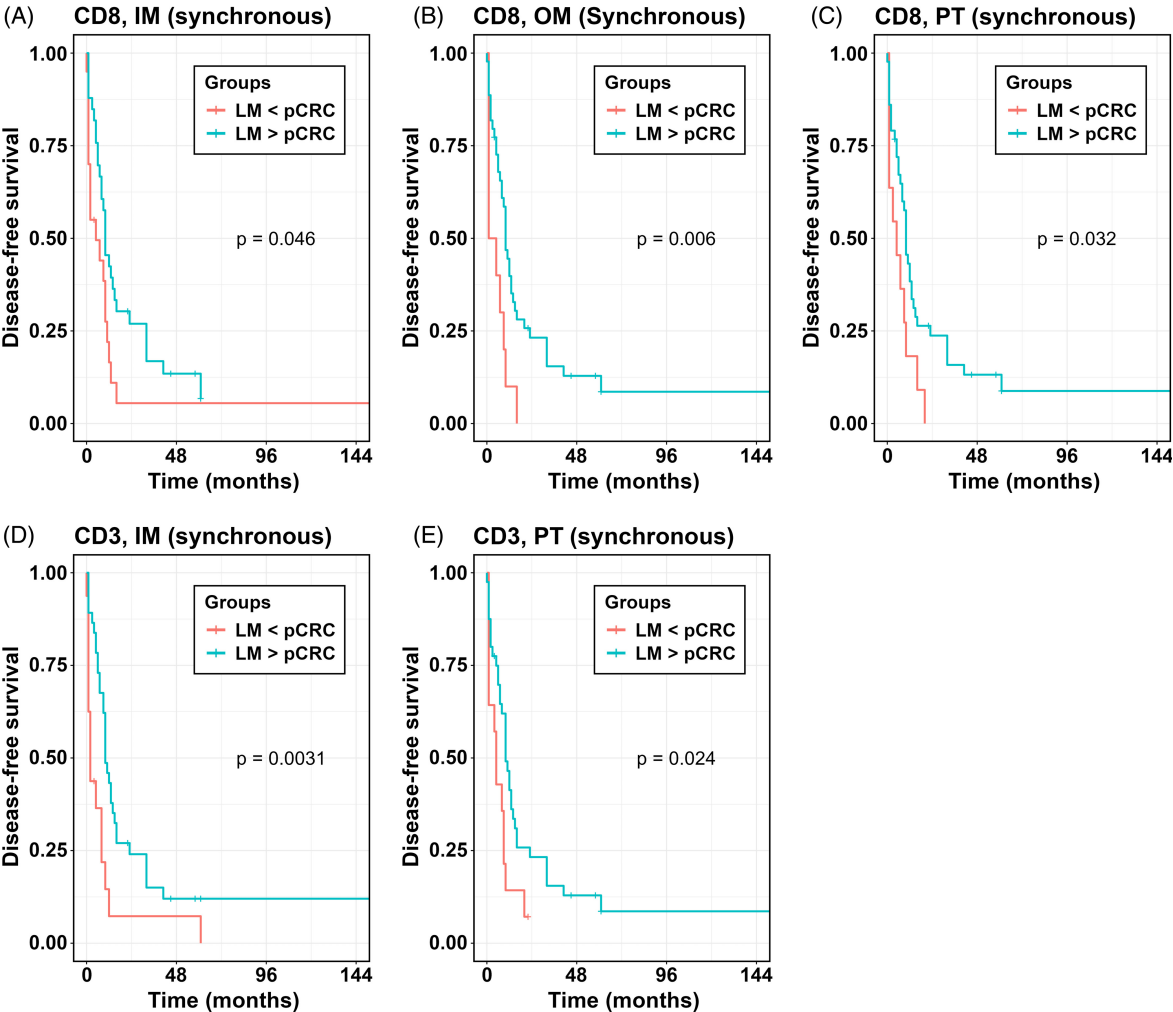
Kaplan–Meier analysis for DFS according to greater T cell densities in specific ROI of LM (LM > pCRC) versus smaller cell densities in specific ROI of LM compared to pCRC (LM < pCRC). *p* values according to log‐rank test. IM, inner invasive margin; LM, liver metastases; OM, outer invasive margin; PT, peritumor zone; ROI, region of interest.

An alternative way of investigating the possible influence of cell densities is to compare survival in individuals with greater cell densities in LM over pCRC (LM > pCRC) versus smaller cell densities in LM (LM < pCRC). For LM > pCRC longer DFS was found only in the synchronous group for CD8+ T cells in IM, OM and PT, and for CD3+ T cells in IM and PT of LM (Figure 3, Table 3). The findings were similar for TTR (Table S9) and OS (Table S10), with the exception for CD45RO+ T cells in TC for which high densities in LM were associated with a borderline increase in risk of death (Table S10).

**TABLE 3:** Hazard ratios for DFS between greater density of T cells in LM compared to pCRC (LM > pCRC) versus smaller density in LM compared to pCRC (LM < pCRC) per individual ROI in CRC patients with synchronous and metachronous metastases.

Cell type and location	Synchronous, *N* = 55		Metachronous, *N* = 44	
	*N* (%)	HR (95% CI), *p*‐value	*N* (%)	HR (95% CI), *p*‐value
CD3 TC	29 (54.7%)	0.64 (0.36–1.16), *p* = 0.140	15 (34.9%)	0.92 (0.46–1.84), *p* = 0.813
CD3 IM	37 (69.8%)	**0.40 (0.21–0.74), *p* = 0.004**	28 (65.1%)	0.64 (0.32–1.25), *p* = 0.192
CD3 OM	51 (94.4%)	0.40 (0.12–1.33), *p* = 0.137	34 (77.3%)	0.75 (0.35–1.61), *p* = 0.463
CD3 PT	40 (74.1%)	**0.47 (0.24–0.91), *p* = 0.024**	30 (71.4%)	0.53 (0.26–1.11), *p* = 0.094
CD8 TC	29 (54.7%)	0.76 (0.43–1.36), *p* = 0.353	16 (37.2%)	1.05 (0.53–2.07), *p* = 0.892
CD8 IM	33 (62.3%)	**0.55 (0.30–1.00), *p* = 0.049**	18 (41.9%)	1.42 (0.71–2.80), *p* = 0.319
CD8 OM	44 (81.5%)	**0.35 (0.17–0.73), *p* = 0.005**	24 (55.8%)	1.24 (0.63–2.45), *p* = 0.540
CD8 PT	43 (79.6%)	**0.47 (0.23–0.94), *p* = 0.032**	27 (62.8%)	0.80 (0.41–1.56), *p* = 0.514
CD45RO TC	27 (52.9%)	1.06 (0.59–1.92), *p* = 0.835	23 (54.8%)	0.84 (0.44–1.62), *p* = 0.600
CD45RO IM	34 (66.7%)	1.01 (0.55–1.87), *p* = 0.977	32 (76.2%)	0.85 (0.40–1.82), *p* = 0.682
CD45RO OM	48 (92.3%)	1.73 (0.53–5.66), *p* = 0.361	36 (85.7%)	0.76 (0.31–1.83), *p* = 0.537
CD45RO PT	43 (82.7%)	0.96 (0.44–2.06), *p* = 0.908	39 (92.9%)	0.60 (0.18–1.97), *p* = 0.397

*Note*: Densities of CD3+, CD8+, and CD45RO+ T cells per area of ROI (mm^2^) were compared between LM and pCRC. Hazard ratios shows the relative risk compared with 1.00 for LM < pCRC group. Bold values indicate statistical significance at the *p* < 0.05 level.

Abbreviations: CI, confidence interval; CRC, colorectal cancer; DFS, disease‐free survival; HR, hazard ratio; IM, inner margin; LM, liver metastases; OM, outer margin; pCRC, primary colorectal cancer; PT, peritumor zone; ROI, region of interest; TC, tumor center.

## 4 DISCUSSION

Increasing evidence suggests that T cells play key roles in anti‐tumor immune response in both pCRC^19^ and in LM,^20^, ^21^, ^36^ and associate with survival. However, comparative data on T cells in tumor microenvironment of pCRC and LM in synchronous and metachronous disease are lacking. Here we show that favorable prognosis was associated with high densities of CD8+ T cells in TC, OM, and PT of LM in the synchronous group only. Comparison between pCRC and LM showed that not only high absolute numbers, but also greater densities of CD8+ T cells in IM, OM, and PT in LM were associated with longer DFS in the synchronous group.

Our study could also show longer survival in patients with metachronous metastases after colon surgery, which associates with greater densities of CD8+ T cells in the TC and IM of pCRC. This may suggest that high densities of effector T cells were contributing to the delay of LM and thus constituted a mechanism distinguishing the metachronous from the synchronous disease.

### 4.1 Prognostic significance of T cells in pCRC and synchronous LM

#### 4.1.1 High densities of CD8+ T cells in LM confer survival benefits

As we have demonstrated, immune cells rather than clinical variables can explain differences in the survival within the synchronous group. Patients, who had high densities of CD8+ T cells in OM and PT of LM, showed longer DFS, which implies that immune cells in the liver around metastases can control their growth. Our results are in accord with previously described prognostic associations for CD8+ T cells in inner margin of LM,^23^ intratumoural CD8+/CD3+ ratios^27^ or immunoscore^20^, ^21^, ^36^ in mixed samples of synchronous and metachronous LM. However, our findings show clear difference in prognostic significance of CD8+ T cells between synchronous and metachronous disease. Of note, LM with the least regressive changes were selected for analysis, which are also the least infiltrated by lymphocytes.^20^ According to findings by Mlecnik and co‐authors, immunoscore in the least‐infiltrated metastasis showed the strongest association with prolonged survival.^20^ Tumor‐infiltrating CD8+ T cells are thought to represent the effector phenotype, which is considered the main anti‐tumor actor in CRC, hepatocellular carcinoma and many other cancers.^37^, ^38^, ^39^

#### 4.1.2 Greater densities of CD8+ T cells in LM over pCRC confer survival benefits

Observed greater densities of effector T cells in OM and PT of LM compared to pCRC at the group level are in line with the concept of higher vulnerability of metastatic tumors to immune response due to lack of sophisticated defensive mechanisms as in the primary tumor.^40^ Greater densities of CD8+ T cells in PT, or CD3+ and CD8+ T cells in tumor margin were observed in synchronous LM versus pCRC in agreement with our results.^41^ However, other authors reported similar densities of CD45RO+ and CD8+,^28^ or only CD8+ T cells,^22^ and significant correlation between pCRC and synchronous LM. Such findings led to hypothesis that evaluation of the local immunity of the metastatic lesion may be substituted by assessment of pCRC.^22^, ^42^ Contrary, Halama et al.,^26^ based on the assessment of CD3+ and CD8+ lymphocytes stated that it was not possible to predict the immunological profile of the hepatic metastases by analyzing only primary tumor lesions. Our findings allow to resolve this ambiguity: densities of CD3+ and CD8+ T cells in all ROI significantly correlated between of pCRC and LM in metachronous group, but were independent in synchronous disease. Therefore prediction of immune microenvironment of LM based on the assessment of pCRC should take into account chronicity status.

When compared between LM and pCRC at individual level, greater densities of CD3+ cells and CD8+ cells in OM of LM versus pCRC were observed in 94% and 82% of patients with synchronous LM, which was significantly more frequent than in the metachronous group (and corresponded to results of correlation analysis). Indeed, only in synchronous group greater densities of CD3+ and CD8+ T cells in specific regions of LM versus pCRC were associated with longer DFS. Detected greater densities of T cells in LM may, on one side, reflect genetic alterations and different antigenicity of tumor cells from primary tumor to metastasis^6^, ^26^ or might be related to specific immune microenvironment of the liver.^6^, ^15^

Fraction of CD8+ T cells out of all T cells in our study corresponds to earlier findings in CRC.^43^, ^44^, ^45^ Since we did not observe any additional associations of CD3+ T cells with survival, all observed effects can be attributed to CD8+ effector T cells. As for clinical value, our results support evaluation of tumor‐infiltrating immune cells at both primary and metastatic sites in order to refine prognosis. In addition, we showed potential to select more patients with favorable prognosis even between those who had lower counts of T cells in synchronous LM. Finally, highlighted implications of T cells into antitumor immunity in CRC reaffirm a utility of personalized T cell‐based immunotherapy.^40^, ^46^

### 4.2 Prognostic significance of T cells in pCRC of metachronous group

Patients with metachronous disease showed significantly longer DFS, TTR, and OS after primary tumor surgery compared to the synchronous group, which corresponds to the literature data.^7^, ^11^ Control of metastases development by microenvironment of primary tumor is known as concomitant immunity.^40^ Worse prognosis in synchronous disease can be due to the loss of such an inhibitory influence after surgical removal of primary tumor. In addition, densities of CD8+ T cells in TC and IM and CD3+ T cells in TC of pCRC were greater in metachronous group. Therefore, cytotoxic T cells through more effective local immunoediting may contribute to delayed occurrence of LM and consequently to longer survival after removal of primary tumor. Smaller density of CD3+ and CD8+ cells in the TC and invasive margin of pCRC in M1 compared to M0 stage patients were observed earlier^46^, ^47^ along with downregulation of immune‐related genes, whereas profile and frequency of mutation in cancer genes were similar. Therefore, our findings confirm the priority of tumor immune microenvironment in defining differences between synchronous and metachronous CRC. Lower counts of CD8+ T cells in the TC and IM of pCRC may identify a group of patients at risk of distant metastasing, who could benefit from thorough monitoring and eventual T cell‐based immunotherapy.^40^, ^46^

### 4.3 Interregional comparisons

Smaller densities of T cells in the tumor interior versus exterior in both pCRC and LM of synchronous and metachronous groups apparently illustrate one of the tumor evasion mechanisms and support using new therapeutics improving lymphocyte infiltration into the tumor.^37^, ^48^, ^49^ Additional source of T cells in OM and PT could be tertiary lymphoid structures, which were frequently observed by us in those ROI, and which are enriched in T cells. Along with beneficial prognostic associations of CD8+ cells in our study, tertiary lymphoid structures have been reported to be associated with a favorable prognosis in CRC and other solid tumors, most likely due to their ability to induce durable antitumor responses.^50^, ^51^ Moreover, since the majority of prognostic associations were found for T cells in PT and OM, current guidelines for assessment of tumor‐infiltrating immune cells in solid tumors may need refinement.^52^

### 4.4 Comparisons with relevant literature

Some preceding studies evaluated tumor‐infiltrating T cells in mixed cohorts of synchronous and metachronous metastases,^27^, ^42^ without strict definition of ROI^23^, ^27^ or in selected microscopic fields of view,^22^, ^28^ which can be a reason for discordant results.

### 4.5 Strengths and limitations of the study

Prognosis in metastatic CRC is driven by metastatic tumor.^3^, ^4^, ^24^ However, which variables (tumor morphology, mutation status, molecular pathways, or immune microenvironment) are responsible for this, remains to be established.^20^, ^45^ To the best of our knowledge no previous studies compared prognostic significance of T cells in pCRC versus paired LM, including separate analysis in synchronous and metachronous patients. Our study design allowed us to demonstrate that local immune status and associated prognostic significance differ between synchronous and metachronous LM and between LM and pCRC. In addition, rigorous definition of ROI, including PT and individual evaluation of IM and OM contributed to obtaining new data as for topography and prognostic significance of T cells in pCRC and LM.

Present study has several limitations, the most important of which we consider the small sample size and missing in many patients data of molecular and mutational analyses. This did not allow us to reliably assess associations of some variables with survival. Survival benefit conferred by CD8+ cells was rather marginal, especially in the TC, as follows from Figure 2, which is attributed to deviant patients with extremely long survival, and again illustrate effect of the sample size. However, we believe this result is solid because it was observed in both Cox regression and Kaplan Meier analyses for DFS and confirmed by related findings for TTR and OS.

The immune infiltrates were investigated in only one metastatic lesion per patient, nevertheless, the least‐infiltrated LM, reported earlier to have the strongest prognostic associations,^20^ were selected. Another limitation is heterogeneous anti‐cancer treatment given in adjuvant and neoadjuvant settings, which did not allow us to obtain reliable data regarding associations between therapy, its effects and T cells. Results would certainly benefit if they go in parallel with assessment of regulatory T cells, innate immune cells and whole exome sequencing data, which is currently ongoing in our laboratory.

## 5 CONCLUSION

We demonstrate here that tumor‐infiltrating T cells contribute to different clinical course and prognosis in patients with synchronous and metachronous CRC. In synchronous CRC, high densities of CD8+ T cells in LM were associated with favorable outcome, and may constitute a prognostic tool in settings where the volume of resected metastatic patients is so large that reliable reference values can be generated. The other key finding of high CD8+ cell densities in TC and IM of pCRC in metachronous CRC may suggest a mechanistic basis for the delay of metastatic spread of malignant CRC cells into the liver. The practical application of this should be possible for a large volume CRC clinic enabling a T‐cell density adjusted risk stratification and follow‐up scheme.

## AUTHOR CONTRIBUTIONS

**Andriy Trailin:** Conceptualization; investigation; writing – original draft; methodology; validation; visualization; writing – review and editing; software; formal analysis; data curation. **Esraa Ali:** Validation; data curation; software; formal analysis; visualization; writing – review and editing; investigation. **Wenjing Ye:** Data curation; formal analysis; software; writing – review and editing; validation; investigation. **Sergii Pavlov:** Data curation; investigation; software; validation; writing – review and editing. **Lenka Červenková:** Data curation; resources; validation; writing – review and editing; investigation; methodology; visualization. **Ondřej Vyčítal:** Data curation; formal analysis; methodology; validation; writing – review and editing; resources; investigation. **Filip Ambrozkiewicz:** Formal analysis; methodology; visualization; writing – review and editing; data curation; validation; investigation. **Petr Hošek:** Visualization; formal analysis; writing – review and editing; validation; methodology. **Ondřej Daum:** Conceptualization; investigation; writing – review and editing; methodology; validation; formal analysis; data curation. **Václav Liška:** Funding acquisition; conceptualization; writing – review and editing; validation; project administration; resources. **Kari Hemminki:** Conceptualization; funding acquisition; methodology; project administration; supervision; writing – review and editing; validation.

## FUNDING INFORMATION

This research was funded by the grant AZV NU21‐03‐00506.

## CONFLICT OF INTEREST STATEMENT

The authors have no conflicts of interest to declare.

## ETHICS STATEMENT

This retrospective study was conducted in accordance with the ethical standards set out in the Declaration of Helsinki (2013 version). The need for informed consent was waived by the Ethics Committee of the Faculty of Medicine and University Hospital in Pilsen, which approved the study (300/2020, June 17, 2020).

## Supporting information


**Data S1:** Supporting Information.

## Data Availability

The de‐identified image data are available on the BioImage Archive and can be accessed via the link: https://www.ebi.ac.uk/biostudies/bioimages/studies/S-BIAD1315. The source code is publicly available on GitHub (https://github.com/sergii01-cuni/script_zones). Further information is available from the corresponding author upon request.
