# Supplementary material for: Prognostic assessment of T‐cells in primary colorectal cancer and paired synchronous or metachronous liver metastasis
Source: Int J Cancer. 2024 Nov 7;156(6):1282–92. doi: 10.1002/ijc.35252 (PMC11736993; doi:10.1002/ijc.35252)
Supplement: Supplementary file 1 — Data S1: Supporting Information. [file IJC-156-1282-s001.pdf]

Prognostic assessment of T-cells in primary colorectal cancer and paired synchronous or metachronous liver metastasis

Andriy Trailin, Esraa Ali, Wenjing Ye, Sergii Pavlov, Lenka Červenková, Ondřej Vyčítal, Filip Ambrozkiewicz, Petr Hošek, Ondřej Daum, Václav Liška, Kari Hemminki

Table of content

Supplementary table 1: Clinical backgrounds of enrolled patients and histopathological features of primary and metastatic tumors

Supplementary table 2: The estimated probability of outcomes for synchronous and metachronous groups in Kaplan-Meier analysis

Supplementary table 3: Sites of recurrence after liver surgery in CRC patients with synchronous and metachronous metastases

Supplementary table 4: Associations of clinical and pathology variables in pCRC and LM with survival (univariable analysis) in CRC patients with synchronous and metachronous metastases

Supplementary table 5: Absolute and relative densities of CD3+ and CD8+ T cells in different ROI of pCRC and LM

Supplementary table 6: Distribution of patients with greater (LM>pCRC) and smaller (LM<pCRC) cell densities in LM compared to pCRC between synchronous and metachronous groups.

Supplementary table 7: Hazard ratios for TTR between high vs. low T cell density per individual ROI of LM in CRC patients with synchronous and metachronous metastases

Supplementary table 8: Hazard ratios for OS between high vs. low T cell density per individual ROI of LM in CRC patients with synchronous and metachronous metastases

Supplementary table 9: Hazard ratios for TTR between greater density of T cells in LM compared to pCRC (LM>pCRC) vs smaller density in LM compared to pCRC (LM<pCRC) per individual ROI in CRC patients with synchronous and metachronous metastases

Supplementary table 10: Hazard ratios for OS between greater density of T cells in LM compared to pCRC (LM>pCRC) vs smaller density in LM compared to pCRC (LM<pCRC) per individual ROI in CRC patients with synchronous and metachronous metastases

Supplementary figure 1: Flow-chart of the project components.

Supplementary figure 2: Survival analysis since colon surgery.

Table S1. Clinical backgrounds of enrolled patients and histopathological features of primary and metastatic tumors

Parameter		Synchronous, N=55	Metachronous, N=44	P value
Age at the diagnosis (years), median (min-max)		62 (29-78)	64 (46-73)	0.31
Gender	male	34 (61.8%)	29 (65.9%)	0.67
	female	21 (38.2%)	15 (34.1%)	
**Primary tumor**				
Location	Right colon	12 (21.8%)	8 (18.2%)	0.65
	Left colon	43 (78.2%)	36 (81.8%)	
Size (cm), median (min-max)		4.3 (1.0-7.5)	3.5 (0.8-8.3)	0.055
Pathologic T stage	T1<br>T2<br>T3<br>T4	0 (0.0%)<br>1 (1.8%)<br>48 (87.3%)<br>6 (10.9%)	1 (2.3%)<br>6 (13.6%)<br>35 (79.5%)<br>2 (4.5%)	0.25
Histological type	NOS<br>Mucinous<br>Other	51 (92.7%)<br>2 (3.6%)<br>2 (3.6%)	39 (88.6%)<br>4 (9.1%)<br>1 (2.3%)	0.27
Pathologic N stage	N0	13(23.6%)	13(29.5%)	0.51
	N1	22 (40.0%)	16 (36.4%)	
	N2	20 (36.4%)	15 (34.1%)	
AJCC 8th staging	Stage I<br>Stage II<br>Stage III<br>Stage IV	 <br><br>55 (100%)	1 (2.3%)<br>12 (27.3%)<br>31 (70.4%)	
Grade	1<br>2<br>3	12 (21.8%)<br>37 (67.3%)<br>6 (10.9%)	14 (31.8%)<br>26 (59.1%)<br>4 (9.1%)	0.77
KRAS status	mutated	17 (30.9%)	7 (15.9%)	0.25
	WT	19 (34.5%)	15 (34.1%)	
	not tested	19 (34.5%)	22 (50.0%)	
BRAF status	mutated	0 (0.0%)	1 (2.3%)	0.19
	WT	24 (43.6%)	13 (29.5%)	
	not tested	31 (56.4%)	30 (68.2%)	
MSI status	instable	1 (1.8%)	1 (2.3%)	0.94
	stable	28 (50.9%)	25 (56.8%)	
	Not tested	26 (47.3%)	18 (40.9%)	
CEA ng/ml		6.8 (1.0-945.0)	8.9 (0.8-1649.0)	0.90
CEA	<5ng/mL	18 (46.2%)	13 (46.4%)	
	>5ng/mL	21 (53.8%)	15 (53.6%)	0.98
Number of examined lymph nodes		13 (1-34)	11 (0-43)	0.38
Lymph node ratio, median (25-75perc)		0.22 (0-0.57)	0.13 (0-0.32)	0.14

Table S1 (continued). Clinical backgrounds of enrolled patients and histopathological features of primary and metastatic tumors

Parameter		Synchronous, N=55	Metachronous, N=44	P value
**Liver metastases**				
Number, median (min-max)		2 (1-24)	1 (1-7)	0.08
Size (cm), median (min-max)		2.0 (0.4-26.0)	2.7 (0.6-7.1)	**0.02**
Grade	1<br>2	21 (39.6%)<br>32 (60.4%)	18 (42.9%)<br>24 (57.1%)	0.76
Metastasis resection<br>R status	R0<br>R1	40 (76.9%)<br>12 (23.1%)	29 (69.0%)<br>13 (31.0%)	0.40
Chemotherapy ± biological regimens	CHT alone<br>CHT + anti-VEGF<br>CHT + anti-EGFR<br>no treatment<br>not known	28 (50.9%)<br>11 (20.0%)<br>9 (16.4%)<br>4(7.3%)<br>3 (5.4%)	30 (68.2%)<br>4 (9.1%)<br>5 (11.4%)<br>2 (4.5%)<br>3 (6.8%)	0.07
Chemotherapy regimens	FOLFOX<br>dle Gramont<br>FOLFIRI<br>others	31 (56.4%)<br>7 (12.7%)<br>4 (7.3%)<br>6 (10.9%)	16 (36.4%)<br>9 (20.5%)<br>2 (4.5%)<br>12 (27.3%)	**0.048**
Chemotherapy ± biological therapy timing	before liver surgery<br>after liver surgery<br>no	37 (67.3%)<br>12 (21.8%)<br>6 (10.9%)	11 (25.0%)<br>32 (72.7%)<br>1 (2.3%)	**0.0001**
Response to preoperative therapy	CR-PR<br>PD-SD	22 (59.5%)<br>15 (40.5%)	4 (40%)<br>6 (60%)	0.27
Response to postoperative therapy	CR-PR<br>PD-SD	1 (8.3%)<br>11 (91.7%)	5 (16.1%)<br>26 (83.9%)	0.5

Notes: Bold values indicate statistical significance at the $p < 0.05$ level.

Abbreviations: NOS: not otherwise specified; AJCC: the American joint committee on cancer; CEA: the carcinoembryonic antigen; CHT: chemotherapy; CR: complete response; PR: partial response; SD: stable disease; PD: progressive disease; MSI: microsatellite instability.

Table S2. The estimated probability of outcomes for synchronous and metachronous groups in Kaplan-Meier analysis

		1y	3y	5y
RFP	synchronous	37.0% (23.9%-50.2%)§	11.9% (2.9%-21.0%)	7.3% (0%-15.5%)
	metachronous	50.0% (35.2%-64.8%)	17.6% (5.8%-29.3%)	Not applicable*
DFS	synchronous	35.4% (22.7%-48.2%)	11.4% (2.7%-20.1%)	7.0% (0%-14.8%)
	metachronous	50.0% (35.2%-64.8%)	17.6% (5.8%-29.3%)	Not applicable*
OS	synchronous	87.2% (78.3%-96.1%)	51.9% (38.3%-65.5%)	31.3% (17.9%-44.8%)
	metachronous	93.2% (85.7%-100.0%)	62.6% (47.7%-77.5%)	34.8% (17.0%–52.7%)

Notes: *there were no complete observations after 5 years

Abbreviations: RFP: recurrence-free proportion; DFS: disease-free survival; OS: overall survival. § estimated probability of outcomes (95% confidence interval)

Table S3. Sites of recurrence after liver surgery in CRC patients with synchronous and metachronous metastases

Site	Synchronous (N, %)	Metachronous (N, %)	P value
Liver	19 (40.4%)	17 (44.7%)	0.689
Lungs	13 (27.7%)	7 (18.4%)	0.318
Liver+lungs	4 (8.5%)	6 (18.8%)	0.300
Colon	2 (4.3%)	2 (5.3%)	0.827
Pelvis	2 (4.3%)	2 (5.3%)	0.827
Other	7 (14.9%)	4 (10.5%)	0.551

Abbreviations: CRC: colorectal cancer.

Table S4. Associations of clinical and pathology variables in pCRC and LM with survival (univariable analysis) in CRC patients with synchronous and metachronous metastases

Variables	Group	Synchronous		Metachronous	
		N (%)	HR (95% CI), p-value	N (%)	HR (95% CI), p-value
**DFS**					
Gender	male	34 (61.8)	1.00	29 (65.9)	1.00
	female	21 (38.2)	0.76 (0.42-1.37), p=0.37	15 (34.1)	1.44 (0.74-2.81), p=0.29
Age	Under median	30 (54.5)	1.00	19 (43.2)	1.00
	Above median	25 (45.5)	0.60 (0.34-1.07), p=0.09	25 (56.8)	0.51 (0.26-0.99), **p=0.046**
Size of pCRC	under median	27 (49.1)	1.00	22 (50.0)	1.00
	above median	28 (50.9)	0.99 (0.56-1.75), p=0.97	22 (50.0)	1.38 (0.71-2.68), p=0.34
Grade of pCRC	Low	48 (88.9)	1.00	40 (90.9)	1.00
	High	6 (11.1)	0.65 (0.25-1.64), p=0.36	4 (9.1)	0.39 (0.09-1.62), p=0.19
Sideness	Right	12 (21.8)	1.00	8 (18.2)	1.00
	Left	43 (78.2)	0.91 (0.46-1.81), p=0.78	36 (81.8)	0.70 (0.31-1.62), p=0.41
N grade	0	13 (23.6)	1.00	13 (29.5)	1.00
	1-2	42 (76.4)	1.25 (0.65-2.42), p=0.50	31 (70.5)	1.18 (0.59-2.34), p=0.65
Size of LM	under median	31 (56.4)	1.00	17 (38.6)	1.00
	above median	24 (43.6)	0.77 (0.43-1.37), p=0.38	27 (61.4)	1.10 (0.56-2.14), p=0.78
Number LM	under median	25 (46.3)	1.00	25 (58.1)	1.00
	above median	29 (53.7)	1.24 (0.70-2.21), p=0.46	18 (41.9)	1.55 (0.79-3.06), p=0.20
Grade of LM	Low	21 (39.6)	1.00	18 (42.9)	1.00
	High	32 (60.4)	0.87 (0.49-1.58), p=0.66	24 (57.1)	1.32 (0.68-2.54), p=0.41
Margin of LM	R0	40 (76.9)	1.00	29 (69.0)	1.00
	R1	12 (23.1)	1.43 (0.73-2.83), p=0.300	13 (31.0)	1.52 (0.74-3.10), p=0.25
**TTR**					
Gender	male	34 (61.8)	1.00	29 (65.9)	1.00
	female	21 (38.2)	0.74 (0.40-1.34), p=0.32	15 (34.1)	1.44 (0.74-2.81), p=0.29
Age	under median	30 (54.5)	1.00	19 (43.2)	1.00
	above median	25 (45.5)	0.59 (0.33-1.06), p=0.08	25 (56.8)	0.51 (0.26-0.99), **p=0.046**
Size of pCRC	under median	27 (49.1)	1.00	22 (50.0)	1.00
	above median	28 (50.9)	0.90 (0.50-1.61), p=0.72	22 (50.0)	1.38 (0.71-2.68), p=0.34
Grade of pCRC	Low	48 (88.9)	1.00	40 (90.9)	1.00
	High	6 (11.1)	0.66 (0.26-1.67), p=0.38	4 (9.1)	0.39 (0.09-1.62), p=0.19
Sideness	Right	12 (21.8)	1.00	8 (18.2)	1.00
	Left	43 (78.2)	0.85 (0.42-1.70), p=0.64	36 (81.8)	0.70 (0.31-1.62), p=0.41

Table S4 (continued). Associations of clinical and pathology variables in pCRC and LM with survival (univariable analysis) in CRC patients with synchronous and metachronous metastases

Variables	Group	Synchronous		Metachronous	
		N (%)	HR (95% CI), p-value	N (%)	HR (95% CI), p-value
N grade	0	13 (23.6)	1.00	13 (29.5)	1.00
	1-2	42 (76.4)	1.19 (0.61-2.30), p=0.61	31 (70.5)	1.18 (0.59-2.34), p=0.65
Median size of LM	under median	31 (56.4)	1.00	17 (38.6)	1.00
	above median	24 (43.6)	0.83 (0.46-1.49), p=0.53	27 (61.4)	1.10 (0.56-2.14), p=0.78
Number of LM	under median	25 (46.3)	1.00	25 (58.1)	1.00
	above median	29 (53.7)	1.26 (0.70-2.26), p=0.44	18 (41.9)	1.55 (0.79-3.06), p=0.20
Grade of LM	Low	21 (39.6)	1.00	18 (42.9)	1.00
	High	32 (60.4)	0.84 (0.46-1.52), p=0.56	24 (57.1)	1.32 (0.68-2.54), p=0.41
Margin of LM	R0	40 (76.9)	1.00	29 (69.0)	1.00
	R1	12 (23.1)	1.53 (0.77-3.04), p=0.22	13 (31.0)	1.52 (0.74-3.10), p=0.25
**OS**					
Gender	male	34 (61.8)	1.00	29 (65.9)	1.00
	female	21 (38.2)	1.55 (0.82-2.93), p=0.18	15 (34.1)	1.55 (0.68-3.54), p=0.30
Age	under median	30 (54.5)	1.00	19 (43.2)	1.00
	above median	25 (45.5)	0.61 (0.33-1.15), p=0.13	25 (56.8)	0.78 (0.34-1.79), p=0.51
Size of pCRC	under median	27 (49.1)	1.00	22 (50.0)	1.00
	above median	28 (50.9)	0.76 (0.41-1.44), p=0.40	22 (50.0)	1.21 (0.53-2.76), p=0.66
Grade of pCRC	Low	48 (88.9)	1.00	40 (90.9)	1.00
	High	6 (11.1)	0.90 (0.35-2.31), p=0.82	4 (9.1)	1.58 (0.37-6.81), p=0.54
Sideness	Right	12 (21.8)	1.00	8 (18.2)	1.00
	Left	43 (78.2)	0.96 (0.45-2.02), p=0.9	36 (81.8)	0.50 (0.19-1.31), p=0.16
N grade	0	13 (23.6)	1.00	13 (29.5)	1.00
	1-2	42 (76.4)	1.34 (0.66-2.68), p=0.41	31 (70.5)	0.67 (0.28-1.60), p=0.37
Median size of LM	under median	31 (56.4)	1.00	17 (38.6)	1.00
	above median	24 (43.6)	0.86 (0.47-1.59), p=0.64	27 (61.4)	0.76 (0.33-1.76), p=0.53
Number of LM	under median	25 (46.3)	1.00	25 (58.1)	1.00
	above median	29 (53.7)	1.60 (0.84-3.07), p=0.16	18 (41.9)	1.87 (0.83-4.18), p=0.13
Grade of LM	Low	21 (39.6)	1.00	18 (42.9)	1.00
	High	32 (60.4)	0.85 (0.44-1.65), p=0.64	24 (57.1)	1.01 (0.45-2.31), p=0.97
Margin of LM	R0	40 (76.9)	1.00	29 (69.0)	1.00
	R1	12 (23.1)	0.97 (0.46-2.05), p=0.93	13 (31.0)	1.61 (0.70-3.71), p=0.27

Notes: Bold values indicate statistical significance at the $p < 0.05$ level. Median age: synchronous = 62, metachronous = 64; median size for primary tumor: synchronous = 4.3 cm, metachronous = 3.5 cm; median size of liver metastasis: synchronous = 2.0 cm, metachronous = 2.7 cm; median number of liver metastasis: synchronous = 2.0, metachronous = 2.1.
Abbreviations: pCRC: primary colorectal cancer; LM: liver metastases; CRC: colorectal cancer; HR: hazard ratio; CI: confidence interval; OS: overall survival.

Table S5. Absolute and relative densities of CD3+ and CD8+ T cells in different ROI of pCRC and LM

	Density of CD3+ T cells	Density of CD3+ T cells	Percentage of CD8+ cells out of CD3+ cells
pCRC TC	461; 11; 2130*	160; 3; 1067	35
pCRC IM	404; 9; 2079	177; 6; 1421	44
pCRC OM	753; 28; 2790	320; 17; 1573	43
pCRC PT	557; 19; 1860	249; 15; 1143	45
LM TC	496; 3; 4000	159; 1; 1507	32
LM IM	777; 2; 5503	220; 1; 1602	28
LM OM	1933; 55; 6403	630; 12; 2726	33
LM PT	1050; 81; 4109	416; 26; 1350	40

*Mean; minimum; maximum

Abbreviations: LM: liver metastases; pCRC: primary colorectal cancer; TC: tumor center; IM: inner margin; OM: outer margin; PT: peritumor zone; ROI: region of interest

Table S6. Distribution of patients with greater (LM>pCRC) and smaller (LM<pCRC) cell densities in LM compared to pCRC between synchronous and metachronous groups.

Cell type and location	Group	synchronous<br>N (%)	metachronous<br>N (%)	P value
CD3 TC	LM<pCRC<br>LM>pCRC	24<br>29 (54.7%)	28<br>15 (34.9%)	0.052
CD3 IM	LM<pCRC<br>LM>pCRC	16<br>37 (69.8%)	15<br>28 (65.1%)	0.625
CD3 OM	LM<pCRC<br>LM>pCRC	3<br>51 (94.4%)	10<br>34 (77.3%)	**0.013**
CD3 PT	LM<pCRC<br>LM>pCRC	14<br>40 (74.1%)	12<br>30 (71.4%)	0.772
CD8TC	LM<pCRC<br>LM>pCRC	24<br>29 (54.7%)	27<br>16 (37.2%)	0.087
CD8 IM	LM<pCRC<br>LM>pCRC	20<br>33 (62.3%)	25<br>18 (41.9%)	**0.046**
CD8 OM	LM<pCRC<br>LM>pCRC	10<br>44 (81.5%)	19<br>24 (55.8%)	**0.006**
CD8 PT	LM<pCRC<br>LM>pCRC	11<br>43 (79.6%)	16<br>27 (62.8%)	0.066
CD45RO TC	LM<pCRC<br>LM>pCRC	24<br>27 (52.9%)	19<br>23 (54.8%)	0.861
CD45RO IM	LM<pCRC<br>LM>pCRC	17<br>34 (66.7%)	10<br>32 (76.2%)	0.314
CD45RO OM	LM<pCRC<br>LM>pCRC	4<br>48 (92.3%)	6<br>36 (85.7%)	0.303
CD45RO PT	LM<pCRC<br>LM>pCRC	9<br>43 (82.7%)	3<br>39 (92.9%)	0.142

Notes: Densities of CD3+, CD8+ and CD45RO+ T cells per area of ROI (mm^2) were compared between LM and pCRC. Bold values indicate statistical significance at the $p < 0.05$ level.
Abbreviations: LM: liver metastases; pCRC: primary colorectal cancer; TC: tumor center; IM: inner margin; OM: outer margin; PT: peritumor zone; ROI: region of interest

Table S7. Hazard ratios for TTR between high vs. low T cell density per individual ROI of LM in CRC patients with synchronous and metachronous metastases

Cell type and location	Synchronous	Metachronous
	HR (95% CI), p-value	HR (95% CI), p-value
CD3 TC	0.81 (0.40-1.65), p=0.565	1.13 (0.56-2.28), p=0.740
CD3 IM	1.17 (0.57-2.38), p=0.668	1.13 (0.54-2.33), p=0.749
CD3 OM	0.79 (0.42-1.46), p=0.444	0.66 (0.30-1.49), p=0.320
CD3 PT	0.78 (0.42-1.45), p=0.426	0.51 (0.22-1.21), p=0.128
CD8 TC	0.61 (0.30-1.24), p=0.172	0.81 (0.40-1.63), p=0.549
CD8 IM	0.75 (0.36-1.55), p=0.438	0.94 (0.48-1.85), p=0.865
CD8 OM	**0.36 (0.18-0.71), p=0.003**	0.81 (0.40-1.64), p=0.564
CD8 PT	**0.30 (0.14-0.63), p=0.002**	0.78 (0.40-1.53), p=0.470
CD45RO TC	0.89 (0.46-1.75), p=0.745	0.82 (0.39-1.71), p=0.598
CD45RO IM	0.94 (0.48-1.84), p=0.853	0.91 (0.44-1.88), p=0.796
CD45RO OM	1.28 (0.67-2.46), p=0.457	0.66 (0.30-1.47), p=0.312
CD45RO PT	1.21 (0.63-2.31), p=0.567	0.94 (0.41-2.14), p=0.874

Notes: Densities of CD3+, CD8+ and CD45RO+ T cells per area of ROI (mm^2) were converted into percentiles and then categorized into low (0–24 percentile) and high (25–100 percentile). Hazard ratios shows the relative risk compared with 1.00 for the low density. Bold values indicate statistical significance at the $p < 0.05$ level. Abbreviations: ROI: region of interest; LM: liver metastases; TTR: time to recurrence; CRC: colorectal cancer; TC: tumor center; IM: inner margin; OM: outer margin; PT: peritumor zone; HR: hazard ratio; CI: confidence interval.

Table S8. Hazard ratios for OS between high vs. low T cell density per individual ROI of LM in CRC patients with synchronous and metachronous metastases

Cell type and location	Synchronous	Metachronous
	HR (95% CI), p-value	HR (95% CI), p-value
CD3 TC	0.51 (0.25-1.04), p=0.063	0.68 (0.29-1.60), p=0.376
CD3 IM	1.06 (0.52-2.14), p=0.870	1.16 (0.45-2.97), p=0.756
CD3 OM	1.17 (0.60-2.29), p=0.646	0.99 (0.37-2.69), p=0.985
CD3 PT	0.85 (0.43-1.68), p=0.647	1.27 (0.43-3.79), p=0.666
CD8 TC	0.57 (0.29-1.13), p=0.107	0.68 (0.28-1.65), p=0.390
CD8 IM	1.00 (0.47-2.12), p=0.997	0.71 (0.31-1.62), p=0.410
CD8 OM	0.75 (0.37-1.50), p=0.410	1.45 (0.54-3.91), p=0.464
CD8 PT	**0.44 (0.21-0.92), p=0.029**	1.62 (0.60-4.36), p=0.342
CD45RO TC	0.82 (0.37-1.80), p=0.619	0.85 (0.32-2.30), p=0.752
CD45RO IM	1.35 (0.62-2.96), p=0.447	1.13 (0.42-3.06), p=0.805
CD45RO OM	0.98 (0.50-1.94), p=0.954	1.01 (0.34-2.99), p=0.981
CD45RO PT	1.54 (0.72-3.27), p=0.262	1.07 (0.36-3.17), p=0.898

Notes: Densities of CD3+, CD8+ and CD45RO+ T cells per area of ROI (mm^2) were converted into percentiles and then categorized into low (0–24 percentile) and high (25–100 percentile). Hazard ratios shows the relative risk compared with 1.00 for the low density. Bold values indicate statistical significance at the $p < 0.05$ level. Abbreviations: ROI: region of interest; LM: liver metastases; OS: overall survival; CRC: colorectal cancer; TC: tumor center; IM: inner margin; OM: outer margin; PT: peritumor zone; HR: hazard ratio; CI: confidence interval.

Table S9. Hazard ratios for TTR between greater density of T cells in LM compared to pCRC (LM>pCRC) vs smaller density in LM compared to pCRC (LM<pCRC) per individual ROI in CRC patients with synchronous and metachronous metastases

Cell type and location	Synchronous	Metachronous
	HR (95% CI), p-value	HR (95% CI), p-value
CD3 TC	0.71 (0.39-1.29), p=0.259	0.92 (0.46-1.84), p=0.813
CD3 IM	**0.45 (0.23-0.88), p=0.019**	0.64 (0.32-1.25), p=0.192
CD3 OM	0.37 (0.11-1.22), p=0.101	0.75 (0.35-1.61), p=0.463
CD3 PT	0.55 (0.27-1.11), p=0.097	0.53 (0.26-1.11), p=0.094
CD8 TC	0.84 (0.46-1.52), p=0.563	1.05 (0.53-2.07), p=0.892
CD8 IM	0.61 (0.33-1.14), p=0.121	1.42 (0.71-2.80), p=0.319
CD8 OM	**0.45 (0.20-0.99), p=0.046**	1.24 (0.63-2.45), p=0.540
CD8 PT	0.58 (0.27-1.21), p=0.147	0.80 (0.41-1.56), p=0.514
CD45RO TC	1.18 (0.65-2.17), p=0.583	0.84 (0.44-1.62), p=0.600
CD45RO IM	0.94 (0.50-1.75), p=0.842	0.85 (0.40-1.82), p=0.682
CD45RO OM	1.67 (0.51-5.45), p=0.398	0.76 (0.31-1.83), p=0.537
CD45RO PT	1.07 (0.47-2.40), p=0.878	0.60 (0.18-1.97), p=0.397

Densities of CD3+, CD8+ and CD45RO+ T cells per area of ROI (mm^2) were compared between LM and pCRC. Hazard ratios shows the relative risk compared with 1.0 for LM<pCRC group. Bold values indicate statistical significance at the $p < 0.05$ level. Abbreviations: ROI: region of interest; pCRC: primary colorectal cancer; LM: liver metastases; CRC: colorectal cancer; HR: hazard ratio; CI: confidence interval; TTR: time to recurrence; TC: tumor center; IM: inner margin; OM: outer margin; PT: peritumor zonw.

Table S10. Hazard ratios for OS between greater density of T cells in LM compared to pCRC (LM>pCRC) vs smaller density in LM compared to pCRC (LM<pCRC) per individual ROI in CRC patients with synchronous and metachronous metastases

Cell type and location	Synchronous	Metachronous
	HR (95% CI, p-value)	HR (95% CI, p-value)
CD3 TC	0.82 (0.43-1.54), p=0.528	0.74 (0.31-1.79), p=0.505
CD3 IM	**0.49 (0.25-0.95), p=0.034**	1.26 (0.49-3.21), p=0.632
CD3 OM	1.00 (0.24-4.22), p=0.998	2.33 (0.69-7.85), p=0.173
CD3 PT	0.95 (0.45-2.00), p=0.883	0.80 (0.32-1.97), p=0.625
CD8 TC	0.84 (0.45-1.58), p=0.592	0.96 (0.42-2.20), p=0.924
CD8 IM	0.78 (0.41-1.49), p=0.447	1.72 (0.77-3.88), p=0.189
CD8 OM	0.80 (0.36-1.76), p=0.577	1.34 (0.59-3.06), p=0.482
CD8 PT	0.81 (0.38-1.72), p=0.584	1.06 (0.46-2.42), p=0.898
CD45RO TC	**2.00 (1.02-3.92), p=0.043**	1.36 (0.59-3.11), p=0.472
CD45RO IM	0.82 (0.41-1.63), p=0.568	1.95 (0.65-5.84), p=0.231
CD45RO OM	1.20 (0.37-3.94), p=0.762	1.06 (0.31-3.59), p=0.927
CD45RO PT	2.18 (0.74-6.41), p=0.156	0.46 (0.10-2.07), p=0.312

Notes: Densities of CD3+, CD8+ and CD45RO+ T cells per area of ROI (mm^2) were compared between LM and pCRC. Hazard ratios shows the relative risk compared with 1.0 for LM<pCRC group. Bold values indicate statistical significance at the $p < 0.05$ level. Abbreviations: ROI: region of interest; LM: liver metastases; pCRC: primary colorectal cancer; CRC: colorectal cancer; HR: hazard ratio; CI: confidence interval; OS: overall survival; TC: tumor cr; IM: inner margin; OM: outer margin; PT: peritumor area.

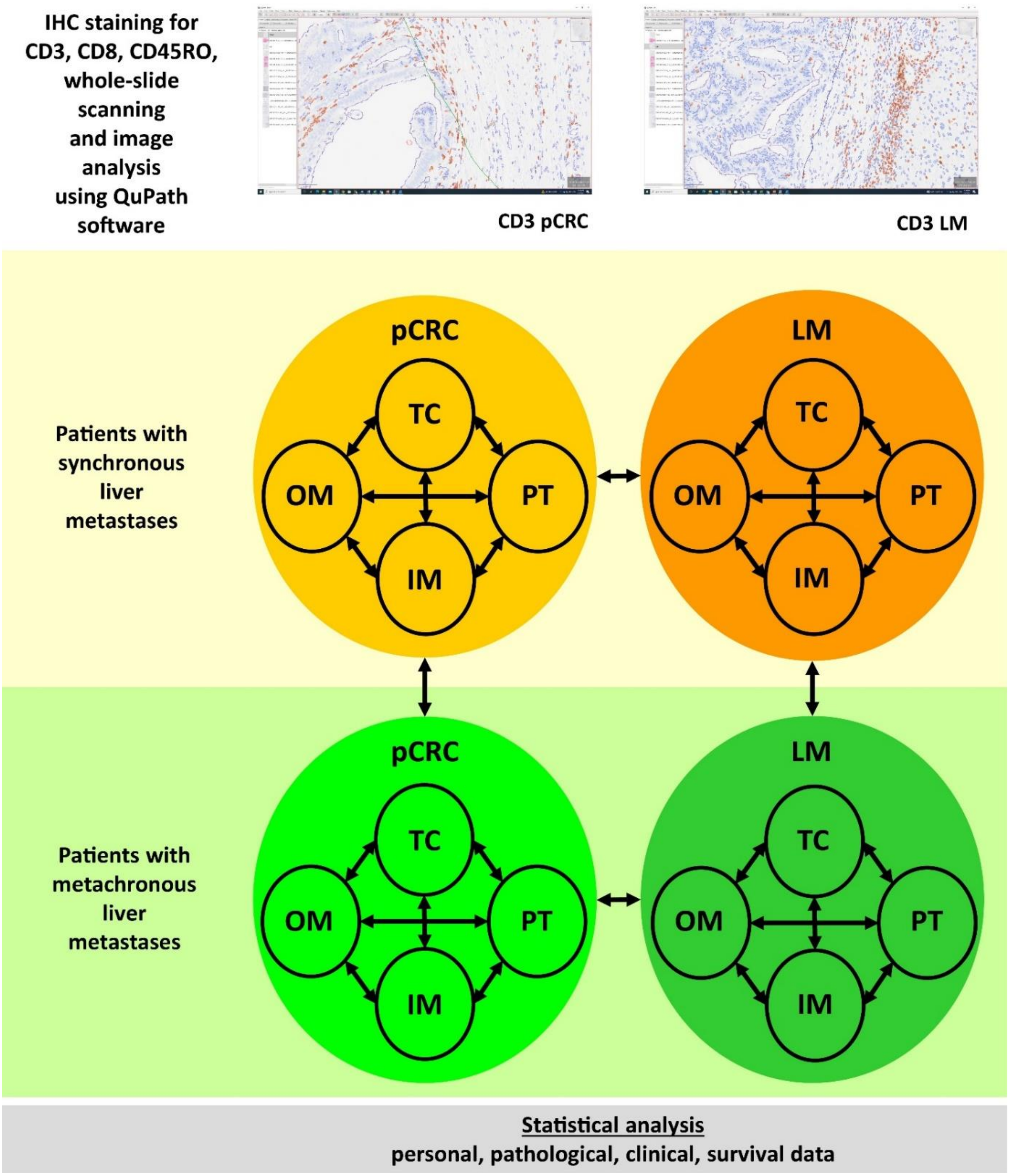

Figure S1. Flow-chart of the project components. Abbreviations: LM: liver metastases; CRC: colorectal cancer; pCRC: primary colorectal cancer; IHC: immunohistochemistry, TC: tumor center; IM: inner margin; OM: outer margin; PT: peritumor zone. Arrows show groups selected for comparisons.

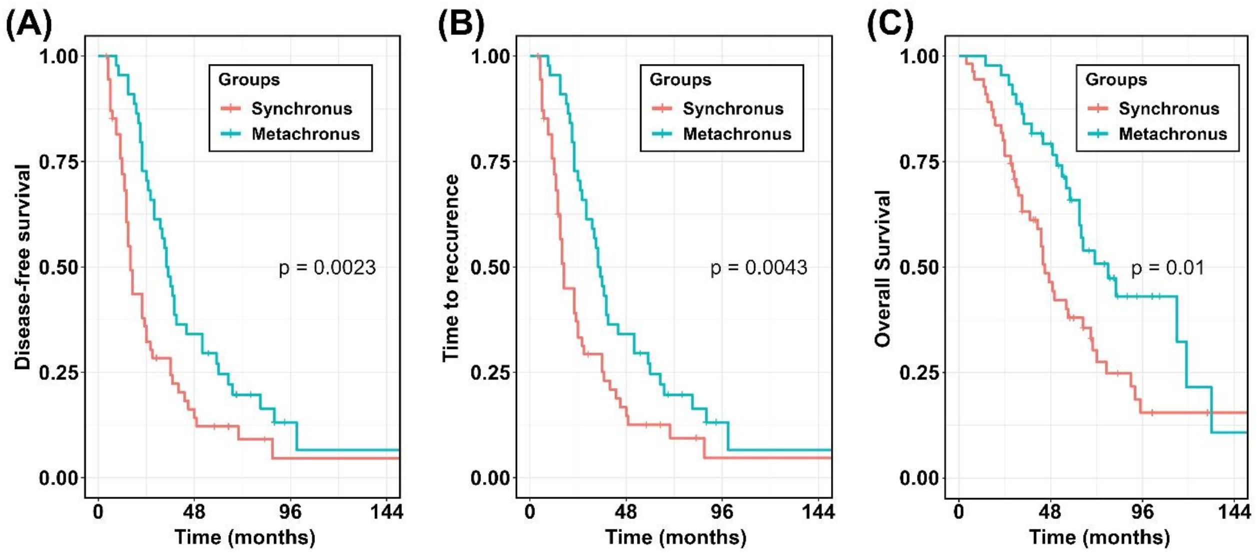

Figure S2. Survival analysis since colon surgery. Up to December 2023, 47 (85.5%) and 42 (76.4%) patients experienced tumour recurrence and had died (P=0.022) in synchronous group, respectively. 38 (86.4%) and 24 (54.5%) patients experienced tumour recurrence and had died in metachronous group, respectively.
